# Describing center of pressure movement in stabilometry by ellipse area approximation

**DOI:** 10.14814/phy2.15390

**Published:** 2022-09-08

**Authors:** Agnieszka Gołąb

**Affiliations:** ^1^ Faculty of Health Sciences Jagiellonian University Medical College Krakow Poland

The paper “A review of center of pressure (COP) variables to quantify standing balance in elderly people: Algorithms and open access code” (Quijoux et al., 2021) states that one of its aims is to give a glossary of variables that are used in stabilometry. However, when describing the movement of COP by ellipse area, the authors mention only the 95% confidence ellipse area. To make the compendium complete it is necessary to list two other methods of ellipse area approximation: a prediction ellipse and its special case—a standard ellipse (Schubert & Kirchner, 2014 and references therein). In a comprehensive study like this, keeping in mind all three of them is essential not only for the completeness of the presentation but mainly because of fundamental differences in meaning and application possibilities of the ellipse area calculated by each method.

The 95% confidence ellipse, the only one mentioned in the discussed review (Quijoux et al., 2021), is defined as the ellipse that with 95% of probability contains the center of the points of the COP sway (generally we can speak about the [1 – α]∙100% of probability but in medical sciences, this value is usually set as 95%). Even if the confidence ellipse covers with a certain probability the unknown population mean it has also the main disadvantage, namely its area cannot be compared with results of investigations carried out according to protocols with different trial duration or sampling frequencies. This is obvious as the confidence ellipse area (CEA) strongly depends on sample size (significantly decreases when the sample size, i.e. the number of the points of the sway, increases) (Rocchi et al., 2005; Schubert & Kirchner, 2014).

The above disadvantage is not the case if a prediction ellipse area (PEA) or a standard ellipse area (SEA) is calculated, as these do not depend on the number of the points of the sway (Duarte, 2015; Rocchi et al., 2005; Schubert & Kirchner, 2014). Generally, the prediction ellipse approximates the region containing a certain percentage of the population, e.g., 95%, i.e. the region where new points (new observations) are located, and its area does not depend on the sample size (Schubert & Kirchner, 2014). It should be also noted that the standard ellipse is nothing else than the special case of the prediction ellipse covering a future point of the COP sway with approximately 63.1% of prediction (Schubert & Kirchner, 2014).

These three ellipses (confidence, prediction, and standard) are centered at the sample mean and have common major and minor axis directions. In my opinion, the parameter of choice in an approximation of COP sway by ellipse areas should be the prediction ellipse area. Also, it should be noted that the way to report the orientation of the ellipse in the medial‐lateral (ML) and anterior–posterior (AP) coordinates has not yet been standardized. Currently, it is done by giving the angle by which the major axis of the ellipse is inclined versus either the ML‐axis (Rocchi et al., 2005) or the AP‐axis (Quijoux et al., 2021). That means that not only the value of the angle should be given but also the information on the convention used. The review paper in question seems to be a pertinent starting point for the standardization of calculation methods used in stabilometry.

## CONFLICT OF INTEREST

The author declares no conflict of interest.
